# Viscosity Factor (VF) Complementary to the Statistical Indicators Associated with the Rheological Behavior of Aqueous Solutions of Polyvinyl Alcohol

**DOI:** 10.3390/polym15071743

**Published:** 2023-03-31

**Authors:** Luis Américo Carrasco-Venegas, José Vulfrano González-Fernández, Luz Genara Castañeda-Pérez, Guido Palomino-Hernández, Federico Alexis Dueñas-Dávila, Salvador Apolinar Trujillo-Pérez

**Affiliations:** 1 Unidad de Posgrado, Facultad de Ingeniería Química, Universidad Nacional del Callao, Bellavista 07011, Peru; 2 Instituto Tecnológico de San Luis Potosí, Tecnológico Nacional de México, San Luis Potosí 78436, Mexico; 3 Facultad de Ciencias Naturales y Matemática, Red Internacional de I+D+i+e de la Escuela de Posgrado RIDIEP, Universidad Nacional Federico Villareal, Lima 15001, Peru; 4 Facultad de Ingeniería Química y Metalurgia, Universidad Nacional de San Cristóbal de Huamanga, Ayacucho 05001, Peru; 5 Facultad de Agronomía, Universidad Nacional Agraria la Molina, Lima 15024, Peru; 6 Facultad de Ciencias e Ingeniería, PELCAN, Pontificia Universidad Católica del Perú, Lima 15088, Peru

**Keywords:** non-Newtonian fluid, rheology, polyvinyl alcohol, differential viscosity

## Abstract

The rheological behavior of aqueous solutions of polyvinyl alcohol at 4, 6, 8 and 10% by weight has been studied and evaluated at temperatures of 20, 25, 30 and 35 °C, using five non-Newtonian fluid models independent of time: Ferrys, Robertson-Stiff, Williamson, Sisko, and Ellis de Haven. The classical method consists in carrying out regression analysis. Using a comparative procedure of determination coefficients and variances, the model that most appropriately adjusts the experimental data to said model is selected. From the statistical point of view, the Sisko and Robertson-Stiff models present better regression parameters; to better specify the choice of the respective rheological model, a new factor has been proposed in the literature, the viscosity factor (VF), which expresses the relationship between apparent and dynamic viscosity. The analysis of this factor for the five models confirms the greater stability of the Ellis de Haven model in terms of the coefficient of variation of the VF. The value of VF fluctuates between 1 and 2 for all ranges of temperature and concentration experienced for vinyl alcohol solutions. As a consequence of the above, for the choice of the non-Newtonian fluid model associated with the rheology of the aqueous solution of polyvinyl alcohol, it is necessary to analyze the statistical parameters and the VF factor simultaneously.

## 1. Introduction

Polyvinyl alcohol (PVA) is a polymer that is the product of the hydrolysis of polyvinyl acetate, which is used in the production of hydrogels, plastics, and biodegradable materials, among many others. According to Singh et al. [1], it is a biodegradable, water-soluble polymer of fossil origin. Due to its biocompatibility, low tendency to bind to proteins, and low toxicity, PVA has wide applications in contact lenses, eye drops, cartilage replacement, and suspension polymerization. Some bacterial species, such as Pseudomonads and Sphingomonads, are known to degrade PVA efficiently. Furthermore, some species of fungi, such as *Penicillium* sp. and *Geotrichum fermentans* WF9101, also degrade PVA. Microbial enzymes such as oxidase, hydrolase, and dehydrogenase play an important role in the degradation of PVA. Arango et al. [2] used a mixture of sericin with polyvinyl alcohol (SS/PVA) for the formation of flexible and potentially biodegradable hydrogels, which their traditional applications were in the pharmaceutical industry and their work researched the use of this biopolymer for agricultural purposes, while its application was limited due to the low stability of soils and high humidity environments. Its properties depend on the molecular weight and the degree of hydrolysis with which it was obtained, and it is soluble in hot water when the degree of hydrolysis of PVA is 90% [3].

It has been described that the viscous behavior can be associated with those materials that do not undergo structural change and those that do experience said change when shear stress is applied, which is determined by the elasticity and the Deborah number (*De*) [4]. In 1964, Reiner [5] introduced the Deborah number in a single-page article as a ratio of relaxation time to observation time, saying the difference between liquids and solids was in the magnitude of *De*. Deborah’s number could be described as a value representing some degree of solidness (a solid material corresponds to a large *De*, while a small *De* corresponds to a liquid material). Zhang et al. [6] presented a study of the rheological properties of shear thickening fluid (STF) when applied as a damper in the automotive industry. Also, they developed a mathematical model to investigate mechanisms of STF-based devices, with which they contribute to the rheology subject.

There are a series of experiences of rheological studies applied to various products; Ramírez [7] presents a detailed theoretical foundation of the aspects related to the rheology of food-oriented fluids. Zargaraan et al. [8] studied the rheological properties of 39 typical Iranian food samples (commercial foods, drinks, and desserts) to classify them as part of diets for people with dysphagia. They determined that the criterion of the apparent viscosity of food is an insufficient characteristic to guarantee the health of patients and proposed including the viscoelasticity parameter as a crucial part of the classification. Pérez et al. [9] conducted experimental tests of Newtonian and non-Newtonian fluids in the Couette viscometer and performed the respective statistical adjustments associated with the Carreu-Yasuda rheological model. In addition, Laurencio et al. [10] obtained the rheological parameters of crude oil associated with the Ostwald de Waele pseudo-plastic model at various temperatures, indicating the importance of determining the apparent viscosity during the treatment and transportation of crude oil.

Additionally, Carrasco et al. [11], using the experimental data from Pérez et al. [9], performed a more detailed statistical treatment that allowed obtaining the parameter “infinite shear rate viscosity”, whose value is not mentioned in the original proposal. Finally, Martínez y Hernández [12] studied the rheology of carbonated sludge; concluded that these materials behave like an ideal plastic.

Other studies have been done by Martínez and Rivera [13], who studied the rheologic behavior of six different commercial brands of Mexican sauces by using a non-conventional vane-in-cup system. Three were considered homogeneous with fine particles, while the others were considered heterogeneous with coarse particles embedded. All of them exhibited a shear-thinning behavior that could be correlated with the power-law model. Dahdouh et al. [14] studied the influence of the high shear rate on rheological behavior, particle size, and other properties of two kinds of orange juices during cross-flow microfiltration. They observed that the shearing condition induces important changes in particle size distribution due to possible fractionation and changes in the rheological properties due to a potential increase of energy of cohesion between the particles. Finally, Yang et al. [15] evaluated the effect of laver powder as a quality factor to improve the rheological properties, texture, and water-holding capacity of *Dosidicus gigas* surimi gel. Their results indicated that adding laver powder increases the hardness, chewiness, and breaking force of this kind of surimi gel.

Furthermore, rheological results indicate that the storage modulus and viscosity of the surimi gel increase when laver powder is added, causing a prolongation of its protein denaturation. In this direction, a recent study with biopolymers concluded that these non-Newtonian fluids were useful for producing various types of food packaging [16]. Furthermore, the study of the rheological properties of non-Newtonian fluids has applications in various fields, such as volcanology [17] y and recently in the area of 3D printing with pseudo polymers by means of a fluid rheology model, in which the parameters that influence the process are detailed: viscosity and oscillatory shear stresses [18].

Additional studies of importance have been carried out by Delmar and Huachum [19], who studied the viscosity of polymer solutions (asphalt binders) using rotational viscometry and determined the flow activation energy from the relationship between apparent viscosity and temperature using the Arrhenius equation. This investigation reveals that the activation energy of flow was different for the various polymer solutions used, where an order of thermal susceptibility can be established based on the magnitude of the activation energy so that this information can be used to predict compaction effort and energy. Furthermore, Carrasco [20] extensively developed the mathematical treatment of non-Newtonian fluids associated with time-independent non-Newtonian fluids, obtaining steady-state and non-steady-state velocity profiles. Finally, Raharja et al. [21] studied applications in the field of polymer injection in the vicinity of extraction wells. They demonstrated that viscosities vary significantly close to the wellbore during polymer injection where flow velocities and, therefore, shear rates are high. In this context, rheological models turn out to be very useful.

The rheological behavior of PVA in an aqueous solution depends on the concentration, temperature, molecular weight, time subjected to shearing, and the magnitude of the shear rate. Therefore, based on experimental data, it is necessary to know how a Newtonian fluid should be characterized from the statistical point of view and its rheological properties. The various types of fluids currently known, of natural or synthetic origin, from the point of view of rheology, are called Newtonian and non-Newtonian; the latter characterizes most known fluids, and their relationship with shear stress and shear rate is not constant. PVA is a non-Newtonian fluid whose rheological behavior is independent of time [22]. In general, rheology is the science that studies the deformation of a fluid when it is subjected to external forces, be it compression or shear forces [23,24]. Investigating the viscosity of materials is one way to evaluate their rheological behavior [25]. Viscosity measurements are of critical importance in a diversity of applications such as food [26], health [27], chemical engineering [28], and pharmacy industries [29], among others.

The importance of its rheological characterization lies in the fact that it allows knowing the rheological model to which the PVA belongs and, at the same time, knowing the parameters of the said model depending on the concentration and temperature; this knowledge is important for the quality control of the products once it has been standardized and for the design of process equipment [30,31].

### 1.1. Properties and Applications of PVA

According to Babayevskii et al. [32], the properties of PVA can be changed reversibly and within wide limits, particularly when the PVA transitions from an elastic gel state to a solid glassy polymer and vice versa. This means that there should be plenty of scope for PVA applications in the production of materials and articles made from it, with its stiffness being decreased, eliminated, or increased through water content.

Another important application is the use of PVA in the production of biomedical materials due to its helpful viscoelastic properties for electrospinning, whose relaxation properties are studied using a tensile load [33]. For example, Zhang et al. [34] mapped PVA and cellulose nanofiber hydrogels using viscoelastic probes. This work proposes an interesting method for probing networks in multi-crosslinked hydrogels. On the other hand, Ueda et al. [35] studied the structural, rheological, and mechanical properties of polyvinyl alcohol composites reinforced with cellulose nanofiber treated with an ultra-high-pressure homogenizer.

The static shear modulus of a methylcellulose solution and the viscoelasticity of a PVA solution at the air/water interface were studied by Abraham et al. [36]. Measurements of the mechanical properties of the surface of a PVA solution and a methylcellulose solution have revealed striking differences. PVA’s response to shear strain is predominantly viscous, whereas methylcellulose is elastic.

Using lignin nanoparticles as nano spacers to adjust the viscoelasticity of a borax-polyvinyl alcohol hydrogel reinforced with cellulose nanofibrils was developed by Bian et al. [37]. Overall, this work demonstrates an easy approach to transferring nanoscale building blocks to 3D polymeric materials with dynamically adjustable rheological properties and may provide a new perspective for the rational design of functional hydrogels for applications requiring high rheological properties. Moud et al. [38] studied the viscoelastic properties of PVA hydrogels with cellulose nanocrystals synthesized by adding sodium chloride, which allowed for verifying the rheological evidence of the formation of the double network.

As can be seen, composite materials that contain PVA as a base are currently being developed. A series of materials are being added to modify their rheological properties. Therefore, it is important to know the properties, as well as the various forms of experimental data treatment of the base material, in this case, PVA, to know to what extent the additions generate changes in the response of the material when applying shear stress.

### 1.2. Viscoelasticity

The classical theory of elasticity considers the mechanical properties of elastic solids by Hooke’s law. That is, the deformation achieved is directly proportional to the applied stress. On the other hand, hydrodynamic theory deals with the properties of viscous liquids. According to Newton’s law, the applied stress is directly proportional to the strain rate but independent of the strain itself. These two categories are idealizations, although the behavior of many solids approximates Hooke’s law (elastic behavior) at infinitesimal strains, and that of many liquids approximates Newton’s law (viscous behavior) at low strain rates. This way, if a force is applied to an elastic solid, it deforms until it ceases, and the deformation returns to its initial value. On the other hand, if stress is applied to a viscous fluid, it deforms but does not recover anything deformed. An intermediate behavior is a viscoelastic behavior in which the body on which the stress is applied recovers part of the applied deformation [39].

To understand a material's rheological behaviour, defining some characteristic times of the material or the experiment is necessary. From the relationship between these times, the behavior will be derived. These tenses are the structural time ($t_{S}$), related to its structure and flow conditions, and is associated with thixotropy; relaxation time (λ), related to the time required for the material to adapt to its new structure and the experimental time ($t_{E}$) related to the time interval in which the suspension is subjected to stress. These parameters are related to each other by the Deborah number mentioned above, the same one defined by: $D_{e}=\lambda /t_{E}$ [40]. According to Bustamante and Aguilera [41], the Deborah number is the main dimensionless number in rheology. One can identify liquid-type or solid-type rheological behaviors for a given material from it.

In general, the rheological behavior of materials can be classified according to the type of flow into two large groups: Flows where there is no structural change, typically Newtonian behavior (constant viscosity), and no thixotropy phenomenon. In turn, you can have two types of materials (Structured: Newtonian fluid with no elasticity. Structured with constant viscosity only at low stresses, which in turn are (a) pure viscous materials, it does not present elasticity in which case $D_{e}<<1$, (b) elastic materials $D_{e}>>1$, and (c) linear viscoelastic materials with $D_{e}\approx 1$). On the other hand, there are materials where there is structural change, typically a non-Newtonian behavior. There are four cases (a) $D_{e}<<1$, $t_{E}>>t_{S}$, does not present thixotropy or elasticity, (b) $D_{e}<<1$, $t_{E}\approx t_{S}$, shows thixotropy but no elasticity, (c) $D_{e}\approx 1$, $t_{E}>>t_{S}$, exhibits nonlinear viscoelasticity, but not thixotropy, (d) $D_{e}\approx 1$, $t_{E}\approx t_{S}$, presents thixotropy but not nonlinear viscoelasticity [42].

In case that $\lambda \ll t_{E}$, Deborah’s number tends to zero, and the rheological behavior developed by the material is of the liquid type, whose most extreme case will be a Newtonian liquid. On the contrary, if $\lambda \gg t_{E}$, Deborah’s number is very large, and the material develops a solid-like behavior, whose asymptotic case will correspond to a Hookean solid. These two extreme behaviors define the ideal constitutive limits for a material, and consequently, the smaller the value of $D_{e}$, the faster the flow of the material will be. When the $D_{e}$ is close to 1, the material will present an intermediate behavior, called viscoelastic. As there is no structural change, the material's response is independent of the applied stress, and therefore, it presents linear viscoelasticity [41].

Normally a graph of the relaxation time expressed as the Deborah number and the strain expressed as the Weisenberg number allows for verifying the rheological characteristic of a material. For example, the material is Newtonian for a low relaxation time and strain value. If a lower deformation load is applied for intermediate relaxation times, the material has the linear viscoelastic behavior represented by models such as Maxwell, Kelvin, and others. If the deformation loads are higher, it presents a nonlinear viscoelastic behavior. For higher relaxation times, but low deformation loads, it exhibits elasticity and for high loads, it exhibits plasticity [39].

An important fact to note is that the linear viscoelasticity equations predict a constant viscosity independent of shear stress. In the same way as Newton’s law, most molten polymers and polymer solutions show a decrease in viscosity with shear rate. For example, at low shears, the interactions between the different polymer chains act as crosslinking points, causing the system’s viscosity to be high. As the shear rate increases, the applied stress causes the destruction of these temporary crosslink points, which leads to a decrease in viscosity. At much higher speeds, the interactions between chains disappear, and the orientation of the different polymer chains according to the flow direction can even be observed.

Williams et al. [43], explain that to relax the tensions, the molecules slide past each other when they are subjected to stress. In addition, they demonstrated how an increase in temperature accelerates stress relaxation. This effect can be explained by the increased free volume between the molecules, allowing them to move more easily and reducing the relaxation time. Therefore, they presented a basic relationship between relaxation time and temperature, known today as the time-temperature superposition principle.

### 1.3. Shear and Extensional Flows

According to Barnes [23], there are two basic types of flow with the relative motion of adjacent liquid particles. They are called shear and extensional flows. In shear flows, liquid elements flow on top of each other, while adjacent elements flow toward or away from each other in extensional flow. It should be noted that all flows offer resistance due to viscosity. Liquids are made to flow by imparting velocity to them. For a given speed, the resultant force increases as the viscosity increases, while for a given force, the speed decreases as the viscosity increases.

Until the 1960s, studies were oriented to shear stress. Since then, interest in extensional flow has increased since it was necessary for many practical situations and because non-Newtonian fluids exhibited very different extensional behavior from those of non-Newtonian fluids. Simple shear flow is the continuous movement of liquid particles over or past each other. Extension or elongation flows are where the liquid particles flow to or from each other [23].

This is a field currently under development that also presents difficulties for its measurement, which is why to date, there are no commercial extensional rheometers available. Its calculation is further complicated because three extensional viscosities must be measured: uniaxial, biaxial, and planar. Münstedt [44] presents an experimental study for the determination of shear and extensional viscosities for a polystyrene sample at 140 °C and shows that the relationship between the elastic modulus E, and the shear modulus G, in the Newtonian region tends to the value of 3, as demonstrated analytically Osswald et al. [39]. In addition, Powell and Housz [45] present extensional viscosity curves as a function of tensile force for various types of thermoplastic polymers.

### 1.4. Yielding Effort

Yield effort $\tau_{0}$ is an important rheological variable in the case of suspensions subjected to relatively low or near-zero shear rates [41]. Normally, the yield stress is considered to have an invariant value for each viscoelastic fluid [46,47,48,49]. However, Barnes et al. [23] introduce a discussion regarding the existence of yield stress, where this rheological parameter is strongly questioned. He also points out that the most popular equations used to describe liquids with yield stress are those of Bingham, Casson, and Herschel-Bulkley since these equations explicitly show the yield stress calculation. While Bustamante and Aguilera [41] conclude that the yield stress is related both to the sensitivity and precision of the viscometer or rheometer used in the experiments carried out at very low shear rates, as well as to the dynamics of the deformation, so this parameter cannot be assigned a unique value.

On the other hand, when the flow curve is analyzed in detail as the shear rate tends to zero, there is a region in which the slope can be considered fixed, so the apparent viscosity in this region tends to a fixed value $\mu_{0}$ and which is called Newtonian viscosity for a very small shear rate. On the other hand, when the shear rate tends to zero, the apparent viscosity tends to infinity, consequently, the values of $\mu_{0}$ tend to be too large so that the slope can be confused with the shear stress axis τ at the beginning of the flow curve, which is often reported as yield stress $\tau_{0}$, although it does not necessarily exist [41].

As a consequence of the above considerations, there are different yield stress values for the same fluid, which implies that there will be as many yield stress values as there are mechanical measurement scenarios involved, defined as the set of contact forces, the rate of application and the conditions under which the solution of PVA and other fluids deforms.

In the present work, the characterization of aqueous solutions of PVA has been carried out, whose rheological characteristic corresponds to a non-Newtonian fluid independent of time through the statistical treatment of the experimental data of shear rate and shear stress; as an additional parameter, the relationship between the apparent viscosity and the differential viscosity is used, with the application of which a more appropriate choice of the model that is associated with the rheological behavior of PVA is estimated.

## 2. Materials and Methods

### 2.1. Rheological Methods Evaluated

The foundations for the rheological characterization of polyvinyl alcohol are the statistical treatment of the shear stress and shear speed data and the calculation of the viscosity factor that involves relating the apparent viscosity and the viscosity for each previously adjusted model.

#### 2.1.1. Nonlinear Least Squares Regression

The least squares regression technique was used on the data of shear stress (τ) and shear rate ($\dot{\gamma }$) to estimate the parameters of the rheological models associated with PVA. This technique consists in finding the parameters that minimize the objective function *S*, given by:

(1) $$S=\sum^{\;}{\left(y_{i}-{\hat{y}}_{i}\right)}^{2}\;$$

(2) $${\hat{y}}_{i}=f\left(x,a,b,\ldots ,n\right)$$

where, $y_{i}$ are observed data of the dependent variable, ${\hat{y}}_{i}$ the projected value of the model. While x expresses the experimental data of the independent variable, f is the equation of the nonlinear model, and a,b,…,n, represent the model parameters. The objective function is minimized, with whose procedure the parameters of the proposed models are found by solving Equation (3), as described by various authors [20].

(3) $$\frac{dS}{da}=\frac{dS}{db}=\ldots =\frac{dS}{dn}=0$$

#### 2.1.2. Rheological Models

The apparent viscosity and the differential viscosity are obtained by conditioning the model. The apparent viscosity is obtained by:

(4) $$\tau =\eta_{ap}\left(\tau ,\dot{\gamma }\right)\times \dot{\gamma }$$

Since τ and $\dot{\gamma }$ are known, the value of the apparent viscosity can be obtained. The differential viscosity is obtained by:

(5) $$\eta_{diff}=\frac{d\tau \left(\tau ,\dot{\gamma }\right)}{d\dot{\gamma }}$$

Table 1 shows the rheological models used and evaluated, considering the incorporation of the new variable.

### 2.2. Evaluation of the Regression and Sensitivity of the VF Criterion

The regression of the models was evaluated with the correlation index statistics $R^{2}$ and variance associated with the regression $S^{2}$ which can be obtained through:

(6) $$R^{2}=1-\frac{\sum_{i=1}^{n}{\left(y_{i}-{\hat{y}}_{i}\right)}^{2}}{\sum_{i=1}^{n}{\left(y_{i}-\overset{-}{y}\right)}^{2}}\;\;\;$$

(7) $$S^{2}=\frac{\sum^{\;}{\left(y_{i}-{\hat{y}}_{i}\right)}^{2}}{N-P}$$

where $\overset{-}{y}$ is the mean of the observed data, N is the number of observed data, and P is the number of model parameters.

For its part, the validity of the model is established through the statistical analysis of the correlation and variance index and the linearity relationship between the apparent viscosity and the differential viscosity; said relationship is proposed to be called the viscosity factor and is given by the equation:

(8) $$\mathrm{VF}=\frac{\eta_{ap}}{\eta_{diff}}$$

Thus, the relationship was studied using the coefficient of variation, which is defined as:

(9) $$\%\mathrm{CV}=\frac{S}{\overset{-}{x}}\times 100$$

(10) $$S=\sqrt{\frac{\sum_{i=1}^{n}{\left(x_{i}-\overset{-}{x}\right)}^{2}}{n-1}}$$

(11) $$\overset{-}{x}=\frac{\sum_{i=1}^{n}x_{i}}{n}$$

where CV is the coefficient of variation, S standard deviation, $\overset{-}{x}$ the average of the analyzed parameter, $x_{i}\;$ are the point values of the analyzed parameter, n data number. On the other hand, the criterion adopted considers that if CV(VF)≤5% analyzed is pertinent and represents its rheological behavior.

### 2.3. Procedure for the Preparation of Experimental Solutions

Viscous solutions of non-hydrolyzed PVA were prepared, CAS No. 9002-89-5 and code number 141351, distributed by the company MERCK-Peru, whose melting point is 240 °C and a density of 670 kg/m^3^ mixing the solid polymer with deionized water from the drinking water deionization plant of the Faculty of Chemical Engineering of the National University of Callao, to obtain polyvinyl alcohol solutions of 4, 6, 8 and 10% by weight at 40 °C to propitiate its solubilization with the help of a magnetic stirrer. Then, the various samples were placed in the sample holder of the rheometer, which is a cylindrical tank provided with a horizontal mark on the inside that indicates the amount of fluid to be used, to which the rotational element called “sipindle” is attached, which in this case is a solid cylinder with a conical end on the underside. The working temperature was stabilized at 25 °C, and then the option that allows for varying the cutting effort was selected, and also the cutting rate was set to 100 s^−1^.

The equipment is started up through the computer, with which the data is collected and stored in a temporary file until the run is completed to 20 °C. Then, the same procedure is continued for 25, 30, and 35 °C for each specified concentration.

**Figure 1:**
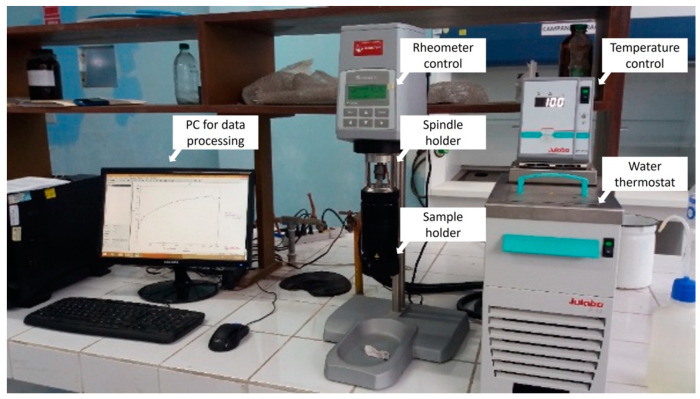
Measuring system—Rheolab QC rotational rheometer. Research laboratory of the Faculty of Chemical Engineering. The National University of Callao. Callao. Peru.

The nonlinear regression analysis was carried out from the data obtained from the rheometer to find the parameters associated with the rheological models the PVA obeys and the correlation index $R^{2}$ and the variance $S^{2}$ and the analysis of variance was applied to the parameters $R^{2}$ and $S^{2}$, to identify the differences in the degree of adjustment of the models studied. With the adjusted data, the viscosity factor was calculated for each of the proposed models. Finally, with the results of the models associated with the PVA and their respective parameters, the shear stress and apparent viscosity rheograms were obtained. With this, the stability criterion of the model was analyzed using the VF. The experimental data were obtained and reported using the Rheolab QC rotational rheometer, as shown in Figure 1.

### 2.4. Computing Environment: Polymath^®^ v6.2 Software

POLYMATH Educational is a computer system created for educational and/or professional purposes. It includes various tools that help users solve mathematical models by applying numerical methods. One of these tools is data processing by correlating them through nonlinear regression. The results are displayed graphically for easy understanding and integration into articles and reports.

## 3. Results

Experimental runs on the rotational rheometer resulted in shear stress rheological data τ(Pa) and cutting rate $\dot{\gamma }$(1/s) for concentrations 4, 6, 8 and 10% of PVA in water at temperatures of 20, 25, 30 and 35 °C, which are shown in Table 2.

Through the Polymath^®^ v6.2 software nonlinear regression was performed with the Ferrys, Robertson-Stiff, Williamson, Sisko, and Ellis de Haven models for each concentration and temperature, being defined by the parameters presented in Table 3. The parameters of the rheological models were calculated together with the correlation index statistics ($R^{2}$) and variance ($S^{2}$) to discern which models best fit the experimental data. In addition, the quotient of apparent viscosity and differential viscosity was calculated to evaluate the trend of this relationship throughout the shear rate interval, whose tendency to a horizontal line is interpreted as a stability criterion in the prediction.

**Figure 2:**
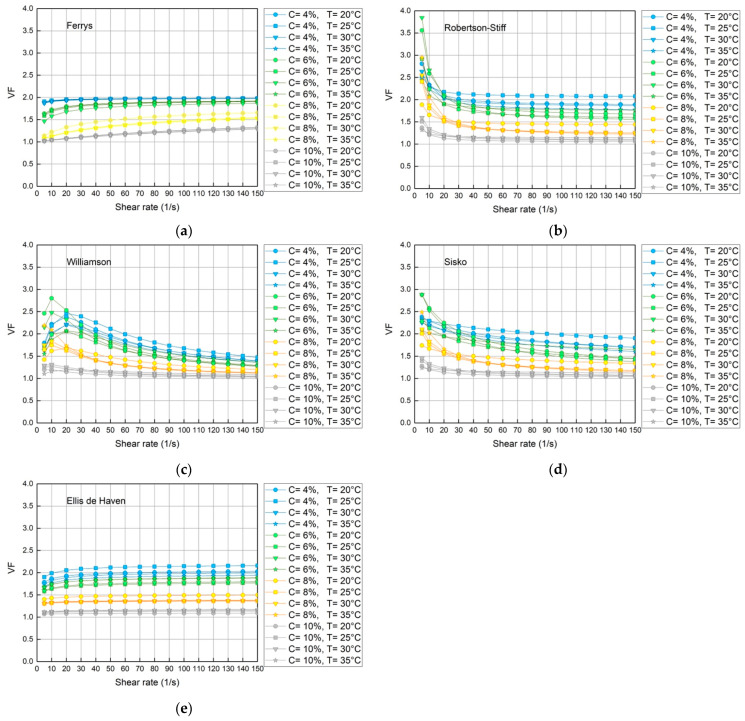
Relationship between apparent viscosity and differences according to evaluated models. The behavior between the apparent and differential viscosity according to the model of (**a**) Ferrys, (**b**) Robertson-Stiff, (**c**) Williamson, (**d**) Sisko, and (**e**) Ellis de Haven.

The graphic results of calculating the viscosity ratio versus the shear rate obtained for each model are shown (see Figure 2). The Robertson-Stiff, Williamson and Sisko models present irregularities concerning the consistency of the VF; however, the Ellis and Ferrys models present a better constant linear projection of the VF for all concentration and temperature ranges. A more detailed analysis of both models through the coefficient of variability confirms the greater stability and reliability of the Ellis model to represent the rheology of polyvinyl alcohol in the range of 4 to 10% in water and at all temperature ranges between 20 and 35 °C, as observed in Table 3.

The ANOVA results for the different models at the level of the parameters $R^{2}$ and $S^{2}$, it can be seen that they are significantly different from each other, showing a significant value at the critical alpha of 0.05 and at 0.01 (see Table 4). Therefore, they are consistent in appropriately modeling the prediction of the rheological properties of vinyl alcohol solutions.

**Figure 3:**
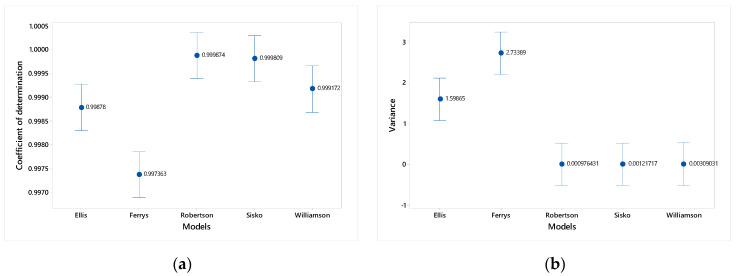
Interval plot for (**a**) the coefficient of determination and (**b**) the variance. 95% confidence interval.

In Figure 3a,b the graphs of intervals are shown in a complementary way for the coefficient of determination and the variance in which, for the first case, values close to 1 for the Robertson-Stiff, Sisko, and Williamson models. Meanwhile, the Ellis and Ferrys models differ slightly from these. On the side of the variance interval graph, an inverse situation can be seen. The Robertson-Stiff, Sisko, and Williamson models have a mean-variance of zero and Ferrys have a mean variance close to 3, while Ellis shows an intermediate variance whose mean value is 1.5.

**Figure 4:**
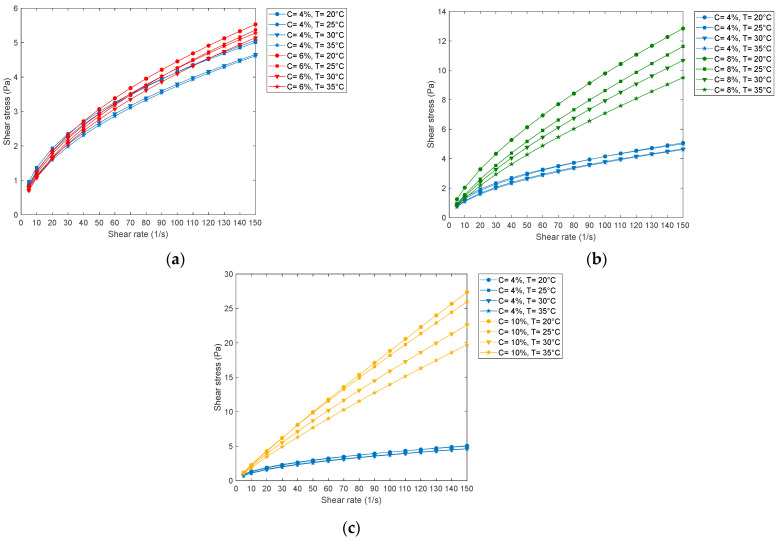
Compared shear stress rheograms for PVA of 4% and of (**a**) 6, (**b**) 8 and (**c**) 10%.

Table 5 shows that the variability coefficient of the VF for the Ellis model is less than 5%, both for the minimum and maximum values. The foregoing agrees with what is expressed in Equation (9), which is why this model is chosen. Then with this model and the parameters found in Table 3, the final comparative rheograms shown in Figure 4 are obtained.

Figure 4a–c shows the comparative rheograms of the shear stress versus the shear rate of 4% PVA with respect to 6, 8 and 10% concentrations. In the rheogram of Figure 4a, a tendency to the characteristics of a real plastic-type fluid is observed at a concentration of 4%. However, for concentrations of 6, 8 and 10% PVA by weight, a pseudo-plastic behavior is observed.

**Figure 5:**
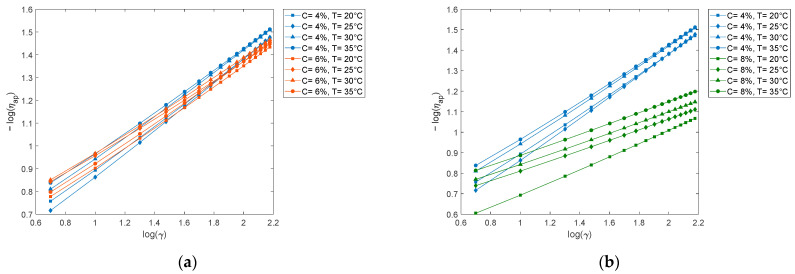

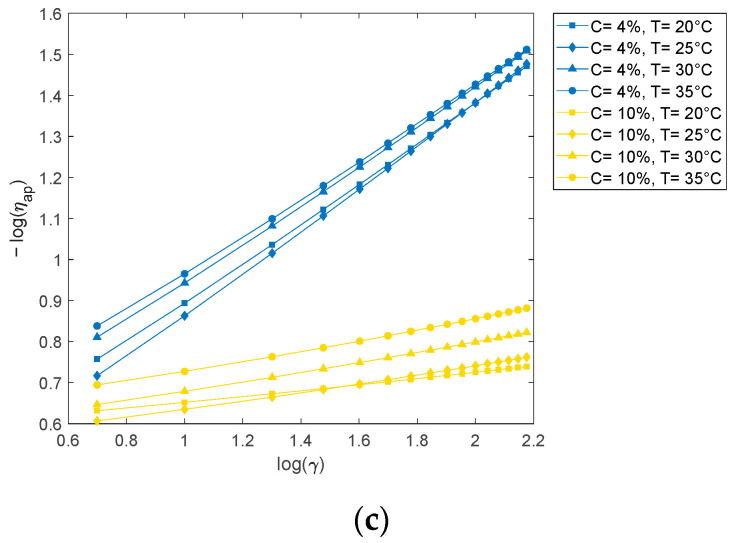
Compared apparent viscosity rheograms for 4% polyvinyl alcohol with respect to (**a**) 6, (**b**) 8, and (**c**) 10%.

Finally, Figure 5a–c shows the rheograms of the apparent viscosity at 4% compared to the concentrations of 6, 8 and 10%. Again, it is observed that for a low cutting rate, it has a highly non-Newtonian behavior for all concentrations; however, when the shear rate increases, the PVA tends to behave like a Newtonian fluid, since the curves become horizontal, as occurs in the apparent viscosity and shear rate rheograms of Newtonian fluids.

## 4. Discussion

According to James [50], when the addition of small amounts of the polymer causes significant changes in a flow, the key fluid property associated with such an effect is extensional viscosity. Extensional viscosity is the analog of shear viscosity, but it is a more difficult property to measure. However, it is possible to apply the CaBER (Capillary breakup extensional rheometry) method to measure this extensional viscosity, which measures the relationship between elongational relaxation and shear relaxation. It is possible to measure the shear viscosity as a function of the shear rate by reaching the stationary regime. However, in extensional flow measurements, steady-state conditions have been achieved only for a few Newtonian and molten fluids, not for any mobile viscoelastic fluids. A steady state in the extension motion should not be expected because the microstructure of the fluid, generally intertwined polymer chains, cannot generally reach an equilibrium configuration in a finite time when the fluid and its microstructure are subjected to constant axial stress. Because the microstructure evolves with time, the normal stresses and extensional viscosity are functions of the strain history.

The evidence pointing to the role of extensional viscosity is partly direct and circumstantial. In the case of laminar flows, its importance is direct. In turbulent flows disturbed by the addition of polymers, mainly boundary layers and jets, the regions most affected by the polymer are zones dominated by shear but periodically subject to significant extensional motion. Therefore, the extensional viscosity is related to the effect. However, in these turbulent flows, simultaneous measurements of the hydrodynamic effect and fluid property have not yet been made, and therefore a direct relationship has not been established. The empirical equations collect all these characteristics summarized in the knowledge of cutting rate and cutting effort, the same ones used in the development of this work.

From the analysis of variance on the correlation index $R^{2}$ It is observed that there are significant differences (p=0) in relation to the degree of fit between the five models studied, where it is highlighted that the Robertson-Stiff model has a higher degree of fit ($R^{2}\to 1$) with respect to others (Figure 3a). Similarly, when parsing the parameter $S^{2}$ where it is observed that there are significant differences between models (p=0), being those of Robertson-Stiff, Sisko, and Williamson the most accepted for having the minimum variance ($S^{2}\to 0$), as seen in Figure 3b. The statistical analysis suggests that the Robertson-Stiff model is the most adequate to represent the rheological behavior of PVA using only statistical criteria; however, these criteria are not enough to choose the definitive model, due to the greater variability of the VF.

The trend of the viscosity factor was analyzed graphically, noting differences in terms of the stability of the predictions, which can be observed in Figure 2a–e, where the order of stability is established (Table 5). Therefore, the Ellis model is considered stable with respect to VF throughout the study interval. From the statistical and graphic analysis, it is concluded that the Ellis model adequately represents the rheological behavior of PVA solutions, although, with respect to the correlation index, the Robertson-Stiff and Sisko models present very high values. close to the unit, and regarding the variance, the Robertson-Stiff, Sisko, and Williamson models present values very close to zero. However, the Ellis model, regarding these parameters, presents acceptable values. In addition, it is observed that the viscosity factor, whose value fluctuates between 1 and 2 in all concentration and temperature ranges, has fewer fluctuations compared to the other models.

The shear stress and shear rate rheograms, based on the Ellis de Haven model, show that the shear stress increases at higher concentrations and at higher temperatures, it decreases (Figure 4a–c). On the other hand, the apparent viscosity rheograms as a function of the shear rate referred to by the Ellis model at concentrations of 6, 8 and 10% of PVA with respect to the concentration at 4% of PVA at all the temperatures experienced are observes a consistently decreasing trend (Figure 5a–c).

Based on Figure 4a–c, it can be seen that at a higher concentration of PVA, considering the same cutting speed, a greater cutting effort is required, and at a higher temperature considering a constant cutting speed, a lower cutting effort is required, which is consistent with numerous experimental data. On the other hand, based on Figure 4a,b, the behavior of the PVA solution in water is pseudoplastic, while at a concentration of 10% of PVA in water, it tends to be an ideal plastic (Figure 4c) for all temperatures.

The proposed method, with more evidence from experimental data to support it, has great potential to choose the most appropriate model among statistically consistent models. In addition, from the point of view of rheological characterization, each of the materials analyzed could be associated with a VF.

## 5. Conclusions

PVA solutions of 4, 6, 8, and 10% were studied at temperatures of 20, 25, 30, and 35 °C, observing that, as the concentration increases, the shear stress and the apparent viscosity increase, and, as the temperature increases, the shear stress values decrease. From the statistical perspective, the models present significant differences with a confidence level of 95%; however, based on the analysis of the statistical parameters (correlation index and variance), the five models can be considered appropriate for predicting the rheological properties of PVA solutions. The research allowed us to postulate a new factor, which has been called the “viscosity factor” (VF), which represents the relationship between apparent viscosity and dynamic viscosity for the characterization of a non-Newtonian fluid. In this case, it has been applied to PVA, whose value fluctuates between 1 and 2 for all ranges of temperature and concentration experienced. VF's statistical consistency and constancy are fulfilled for the Ellis de Haven model, while for the other models, this factor presents a greater variability.

## Figures and Tables

**Table 1:** The rheological model evaluated in the framework of the study.

Model	Ferrys	Robertson-Stiff	Ellis de Haven	Williamson	Sisko
Equation	$\tau =\frac{A}{1+\frac{\tau }{B}}\dot{\gamma }$	$\tau =A{\left(B+\dot{\gamma }\right)}^{n}$	$\tau =\frac{A\dot{\gamma }}{1+B\tau^{n-1}};\;n>1$	$\dot{\gamma }=\frac{\tau }{\frac{A}{B+\dot{\gamma }}+C}$	$\tau =A\dot{\gamma }+B{\dot{\gamma }}^{n}$
Apparent viscosity	$\frac{AB}{B+\tau }$	$A{\left(B+\dot{\gamma }\right)}^{n}{\dot{\gamma }}^{-1}$	$\frac{A}{1+B\tau^{n-1}}$	$\frac{A}{B+\dot{\gamma }}+C$	$A+B{\dot{\gamma }}^{n-1}$
Differential viscosity	$\frac{AB}{B+2\tau }$	$An{\left(B+\dot{\gamma }\right)}^{n-1}$	$\frac{A}{1+Bn\tau^{n-1}}$	$\frac{A\left(B+\dot{\gamma }\right)-A\dot{\gamma }}{{\left(B+\dot{\gamma }\right)}^{2}}+C$	$A+Bn{\dot{\gamma }}^{n-1}$

**Table 2:** Rheology Data for Polyvinyl Alcohol 4, 6, 8 and 10%.

	T 20 °C		T 25 °C		T 30 °C		T 35 °C	
	$\dot{\gamma }$ **(1/s)**	τ **(Pa)**	$\dot{\gamma }$ **(1/s)**	τ **(Pa)**	$\dot{\gamma }$ **(1/s)**	τ **(Pa)**	$\dot{\gamma }$ **(1/s)**	τ **(Pa)**
4% of PVA in water	4.63	1	4.47	1	6.61	1	6.91	1
	12.8	1.5	10.6	1.5	15.7	1.5	17.4	1.5
	23.3	1.99	21.4	1.99	28.8	1.99	30.8	1.99
	36.7	2.49	34.5	2.49	44.5	2.49	46.7	2.49
	52.6	2.99	50.5	2.99	62.8	2.99	65.5	2.99
	70.8	3.49	69.2	3.49	84.1	3.49	86.9	3.49
	91.4	3.98	91.2	3.98	-	-	-	-
6% of PVA in water	3.23	1	6.41	1	5.32	1	5.99	1
	12.5	1.5	15.6	1.5	15.7	1.5	14.6	1.5
	22.5	1.99	27.1	1.99	28.4	1.99	25.1	1.99
	34.9	2.49	40.2	2.49	43	2.49	37.4	2.49
	48.8	2.99	54.5	2.99	58.8	2.99	52.2	2.99
	64.5	3.49	70.7	3.49	75.7	3.49	69	3.49
	81.3	3.98	87.8	3.98	93.5	3.98	88.1	3.98
	99.9	4.48	-	-	-	-	-	-
8% of PVA in water	2.78	1	3.88	1	3.75	1	3.48	1
	6.45	1.745	6.49	1.5	7.51	1.5	9.45	1.5
	12.1	2.49	12.4	1.99	14.1	1.99	16.4	1.99
	16.7	2.99	17.8	2.49	20.2	2.49	23.8	2.49
	21.5	3.49	23.6	2.99	26.4	2.99	31.1	2.99
	26.5	3.98	29.5	3.49	33.1	3.49	38.6	3.49
	31.8	4.48	35.6	3.98	40.3	3.98	46.6	3.98
	37.0	4.98	41.9	4.48	47.1	4.48	54.7	4.48
	42.6	5.48	48.8	4.98	53.9	4.98	62.9	4.98
	48.3	5.97	55.6	5.48	61.2	5.48	71.0	5.48
	54.1	6.47	61.9	5.97	68.5	5.97	79.5	5.97
	60.3	6.97	68.4	6.47	76.0	6.47	87.7	6.47
	66.9	7.47	75.4	6.97	83.6	6.97	96.0	6.97
	73.4	7.96	82.1	7.47	91.4	7.47	-	-
	80.1	8.46	88.8	7.96	99.1	7.96	-	-
	86.9	8.96	95.9	8.46	-	-	-	-
	93.7	9.46	-	-	-	-	-	-
10% of PVA in water	2.7	1	1.94	1	2.07	1	4.5	1
	7.1	1.745	4.78	1.5	5.43	1.5	7.01	1.5
	10.9	2.49	8.2	1.99	9.13	1.99	10.4	1.99
	13.3	2.99	10.4	2.49	12	2.49	13.0	2.49
	15.7	3.49	12.3	2.99	14.5	2.99	16.7	2.99
	18.1	3.98	15.1	3.49	17.2	3.49	19.9	3.49
	20.9	4.48	18.1	3.98	20.2	3.98	23.3	3.98
	23.7	4.98	20.7	4.48	23.4	4.48	26.9	4.48
	26.4	5.48	23.4	4.98	26.2	4.98	30.7	4.98
	29	5.97	26.0	5.48	29.3	5.48	34.3	5.48
	31.7	6.47	28.6	5.97	32.6	5.97	37.3	5.97
	34.4	6.97	31.2	6.47	35.9	6.47	40.8	6.47
	37.1	7.47	34.0	6.97	39.2	6.97	44.9	6.97
	39.7	7.96	36.8	7.47	42.3	7.47	48.6	7.47
	42.3	8.46	39.5	7.96	45.7	7.96	52.7	7.96
	45.0	8.96	42.7	8.46	49	8.46	55.8	8.46
	47.7	9.46	45.5	8.96	52.4	8.96	59.8	8.96
	50.1	9.95	48.1	9.46	55.6	9.46	64.3	9.46
	52.8	10.5	51.1	9.95	58.9	9.95	67.7	9.95
	55.5	10.9	53.7	10.5	62.2	10.5	71.5	10.5
	58.3	11.4	56.9	10.9	65.4	10.9	76.1	10.9
	60.9	11.9	59.5	11.4	68.7	11.4	79.4	11.4
	63.7	12.4	62.5	11.9	72.1	11.9	83.3	11.9
	66.4	12.9	65.6	12.4	75.5	12.4	87.6	12.4
	69.1	13.4	68.4	12.9	78.9	12.9	90.8	12.9
	71.8	13.9	71.2	13.4	82.4	13.4	95.2	13.4
	74.8	14.4	74.4	13.9	85.8	13.9	98.9	13.9
	77.5	14.9	77.1	14.4	89.2	14.4	-	-
	80.4	15.4	79.9	14.9	92.5	14.9	-	-
	83.3	15.9	82.9	15.4	95.8	15.4	-	-
	86.0	16.4	85.7	15.9	99.3	15.9	-	-
	88.9	16.9	88.8	16.4	-	-	-	-
	91.7	17.4	91.9	16.9	-	-	-	-
	94.5	17.9	94.9	17.4	-	-	-	-
	97.3	18.4	98	17.9	-	-	-	-

**Table 3:** Ferrys, Robertson-Stiff, Williamson, Sisko, and Ellis de Haven parameters.

		4% of PVA in Water				6% of PVA in Water				8% of PVA in Water				10% of PVA in Water			
		T 20 °C	T 25 °C	T 30 °C	T 35 °C	T 20 °C	T 25 °C	T 30 °C	T 35 °C	T 20 °C	T 25 °C	T 30 °C	T 35 °C	T 20 °C	T 25 °C	T 30 °C	T 35 °C
Ferrys parameters	*A*	1.664	1.948	1.254	1.318	0.460	0.371	0.255	0.410	0.242	0.159	0.148	0.135	0.247	0.240	0.205	0.180
	*B*	0.107	0.093	0.119	0.108	0.475	0.536	0.776	0.491	6.560	9.963	9.042	7.636	51.500	51.500	51.500	46.505
	$R^{2}$	0.999	0.998	0.999	0.999	0.997	0.996	0.996	0.999	0.998	0.995	0.996	0.994	0.996	0.998	0.999	0.999
	$S^{2}$	0.711	3.081	0.668	1.674	3.486	3.862	5.631	0.597	1.834	4.443	4.582	6.035	3.493	1.702	1.207	0.738
Robertson-Stiff parameters	*A*	0.3457	0.441	0.309	0.260	0.248	0.217	0.175	0.3005	0.384	0.186	0.168	0.128	0.228	0.246	0.224	0.241
	*B*	2.5550	1.103	2.150	3.402	6.036	4.389	7.989	2.0124	1.621	4.940	5.565	7.674	1.535	2.025	2.339	0.795
	*n*	0.5380	0.485	0.543	0.576	0.620	0.642	0.676	0.5746	0.702	0.826	0.829	0.859	0.955	0.930	0.921	0.879
	$R^{2}$	1.0000	0.999	0.999	1.000	0.999	1.000	1.000	0.9999	0.999	0.999	0.999	0.999	1.000	0.999	0.999	0.999
	$S^{2}$	-	0.0006	0.0002	-	0.0001	0.0001	0.0001	0.0002	0.001	0.002	0.001	0.0002	0.001	0.002	0.002	0.002
Williamson parameters	*A*	1.687	1.897	1.590	1.473	1.445	1.412	1.273	1.684	2.562	1.485	1.371	1.136	0.873	1.275	1.266	1.930
	*B*	4.774	5.378	6.131	5.667	2.271	5.181	2.890	6.807	7.278	3.864	3.304	1.750	4.973	5.350	5.073	16.85
	*C*	0.027	0.024	0.024	0.025	0.032	0.031	0.030	0.028	0.076	0.074	0.068	0.061	0.181	0.171	0.149	0.124
	$R^{2}$	0.998	0.999	0.999	0.999	0.997	0.999	0.999	0.998	1.000	1.000	1.000	1.000	1.000	1.000	1.000	1.000
	$S^{2}$	0.003	0.002	0.001	0.001	0.007	0.001	0.001	0.003	0.004	0.001	0.002	0.001	0.004	0.008	0.008	0.002
Sisko parameters	*A*	0.012	0.007	0.010	0.013	0.023	0.020	0.023	0.010	0.044	0.064	0.059	0.057	0.174	0.161	0.138	0.087
	*B*	0.515	0.533	0.431	0.429	0.642	0.436	0.564	0.409	0.553	0.539	0.541	0.613	0.260	0.372	0.383	0.226
	*n*	0.384	0.408	0.412	0.387	0.274	0.369	0.258	0.451	0.500	0.321	0.297	0.203	0.390	0.390	0.379	0.683
	$R^{2}$	1.000	1.000	1.000	1.000	0.999	1.000	1.000	1.000	1.000	1.000	1.000	1.000	1.000	1.000	1.000	1.000
	$S^{2}$	0.000	0.000	0.000	0.000	0.002	0.000	0.000	0.001	0.001	0.002	0.001	0.000	0.002	0.004	0.004	0.002
Ellis de Haven parameters	*A*	0.631	0.744	0.511	0.463	0.592	0.473	0.456	0.548	0.946	0.712	0.648	0.567	0.437	0.461	0.452	0.351
	*B*	3.020	3.020	3.020	3.020	3.020	3.020	3.020	3.020	2.497	3.020	3.020	3.020	0.851	0.822	0.974	0.735
	*n*	2.089	2.213	2.062	2.006	1.939	1.831	1.859	1.945	1.546	1.407	1.417	1.430	1.150	1.216	1.231	1.276
	$R^{2}$	0.999	0.999	0.999	0.999	0.998	0.999	0.997	1.000	0.999	0.998	0.998	0.997	1.000	1.000	1.000	1.000
	$S^{2}$	1.198	0.935	1.239	1.590	3.681	1.910	5.494	0.581	0.645	1.981	2.057	3.183	0.142	0.321	0.405	0.215

**Table 4:** Analysis of variance for the coefficient of determination ($R^{2}$ ) and variance ($S^{2}$ ) according to models.

Parameter	Source	DF	SS	MS	F	*p*-Value
ANOVA for $R^{2}$	Models	4	0.000067	0.000017	17.28	0.000
	Error	75	0.000073	0.000001		
	Total	79	0.000139			
ANOVA for $S^{2}$	Models	4	100.26	25.066	22.75	0.000
	Error	75	82.65	1.102		
	Total	79	182.91			

DF: Degrees of freedom; SS: Sum of squares between treatments; MS: Mean square of the factor; F: Fisher’s probability value.

**Table 5:** Stability in prediction based on VF.

Model	%CV Minimum	%CV Average	%CV Maximum	Stability	Order
Ferrys	1.04	6.12	10.49	NO	2
Ellis	0.47	2.26	3.65	YES	1
Robertson-Stiff	3.90	15.26	31.82	NO	5
Sisko	2.89	12.78	26.07	NO	3
Williamson	3.25	14.95	27.57	NO	4

## Data Availability

Not applicable.
